# Free boundary minimal Möbius bands in toroids

**DOI:** 10.1007/s00526-026-03382-6

**Published:** 2026-06-23

**Authors:** Mario B. Schulz

**Affiliations:** Dipartimento di Matematica, Università di Trento, via Sommarive 14, Povo, 38123 Trento Italy

**Keywords:** 53A10, 58E12, 49Q20

## Abstract

We prove that round, strictly mean convex toroids of revolution contain infinitely many (geometrically distinct) embedded free boundary minimal Möbius bands as well as infinitely many embedded free boundary minimal annuli. The surfaces in both families are constructed by means of equivariant variational methods and their areas grow linearly with the order of their symmetry groups.

## Introduction

Given a compact, three-dimensional Riemannian manifold *M* with nonempty boundary ∂M, can a compact surface of a given topological type be realised as free boundary minimal surface embedded in the ambient manifold *M*? A solution $\Sigma \subset M$ of this problem is a critical point for the area functional among all surfaces in *M* with boundary constrained to ∂M. Equivalently, Σ has vanishing mean curvature and meets the ambient boundary ∂M orthogonally along its own boundary. The existence question is open in general and particularly challenging when aiming for unstable critical points. In such cases, min-max methods offer promising avenues for existence results. The min-max theory for the area functional was pioneered by Almgren [1] and Pitts [37], and later significantly advanced by Marques–Neves [28, 29], leading to substantial progress in minimal surface theory. For the construction of free boundary minimal surfaces with prescribed topology, it is advantageous to employ the min-max theory by Simon–Smith [40] and Colding–De Lellis [7], for which topological control results are available. Min-max methods have indeed been successful in constructing embedded free boundary minimal surfaces of various topological types [3, 12, 17, 23, 25, 39].

Controlling the topology of the resulting minimal surface is the main difficulty in the min-max approach. In the setting of Simon–Smith min-max theory, general genus bounds have been obtained in [10, 24, 25]. The author’s joint work with Franz [12] also provides a general result providing control on the number of boundary components of a free boundary minimal surface obtained through min-max methods. The main result [12, Theorem 1.8] is applicable in ambient manifolds with strictly mean convex boundary and establishes lower semicontinuity of the first Betti number along min-max sequences. Notably, the theorem does not require the assumption of orientability. Nonorientable surfaces cannot be properly embedded in simply connected ambient 3-manifolds such as the Euclidean unit ball for topological reasons. The goal of this article is to demonstrate that certain three-dimensional ambient manifolds with mean convex boundary and nontrivial topology do contain infinitely many embedded free boundary minimal Möbius bands. The inspiration for this work dates back over 80 years to the historical origins of the field. Indeed, Courant [9, II. § 3.2] wrote that “the most interesting problems with free boundaries are those in which the entire boundary is free on a given closed surface *not* of genus zero, e. g. on a *torus*.”

We consider the compact toroid $M^{\rho }$ in $\mathbb {R}^3$ bounded by a rotationally symmetric torus with outer radius ρ>2 and unit circular vertical cross-sections as ambient manifold. More precisely,

1 $$\begin{aligned} M^{\rho }=\Bigl \{(x_1,x_2,x_3)\in \mathbb {R}^3\,{:}\,\bigl (\sqrt{x_1^2+x_2^2}-\rho \bigr )^2+x_3^2\le 1\Bigr \}. \end{aligned}$$

The assumption ρ>2 implies that the boundary $\partial M^{\rho }$ is smooth and strictly *mean convex*. Indeed, the sum of the principal curvatures $\kappa _1=1$ and $\kappa _2\ge -1/(\rho -1)$ is positive if ρ>2. Mean convexity is an important condition, ensuring that embedded free boundary minimal surfaces Σ in $M^{\rho }$ are necessarily properly embedded, i. e. embedded and satisfying $\partial \Sigma =\Sigma \cap \partial M^{\rho }$.

**Fig. 1:**
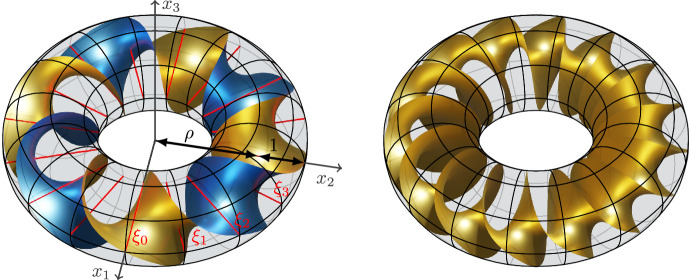
$\mathbb {D}_n$-equivariant free boundary minimal surfaces $\mathcal {O}_n$ in $M^{\rho }$ for ρ=2.2 and $n\in \{8,15\}$

Given $n\in \mathbb {N}$, we understand the *dihedral group* $\mathbb {D}_n$ of order 2*n* as the subgroup of Euclidean isometries acting on $M^{\rho }$ generated by the rotation $\textsf {R}_{e_1}^{\pi }$ of angle π around the $x_1$-axis and by the rotation $\textsf {R}_{e_3}^{2\pi /n}$ of angle 2π/n around the $x_3$-axis. Being generated by rotations, the dihedral group is orientation-preserving and contains the cyclic group $\mathbb {Z}_n$ as a subgroup. The dihedral group has already proven extremely useful for equivariant min-max constructions in the unit ball [3, 12] and in ellipsoids [39]. For each $k\in \{0,\ldots ,2n-1\}$ we define the horizontal segment

2 $$\begin{aligned} \xi _k:=\Bigl \{\Bigl (r\cos \Bigl (\frac{\pi k}{n}\Bigr ),r\sin \Bigl (\frac{\pi k}{n}\Bigr ),0\Bigr )\,{:}\, r\in [\rho -1,\rho +1]\Bigr \} \end{aligned}$$

as visualised in Fig. 1 (left image). The union $\xi _0\cup \ldots \cup \xi _{2n-1}$ is fixed under the action of the dihedral group $\mathbb {D}_n$ and $\mathbb {D}_n$ contains the rotation of angle π around $\xi _k$ for any $k\in \{0,\ldots ,2n-1\}$. We prove the following existence result.

### Theorem 1.1

For every ρ>2 and every integer $n\ge 5(\rho +1)/2$ the toroid $M^{\rho }$ defined in (1) contains an embedded, $\mathbb {D}_n$-equivariant free boundary minimal surface $\mathcal {O}_n$ with the following properties.

(i) $\mathcal {O}_n$ contains the segment $\xi _k$ for all even *k* and intersects it orthogonally for all odd *k*.
(ii) $\mathcal {O}_n$ is a topological Möbius band if *n* is odd respectively an annulus if *n* is even.
(iii) The area of $\mathcal {O}_n$ is strictly between nπ and 2nπ.

Theorem 1.1 reveals striking differences between free boundary minimal surfaces in toroids when compared to those in the Euclidean unit ball. Indeed, Toro Cardona [41] proved that even in the class of branched minimal immersions, the 3-dimensional Euclidean unit ball $\mathbb {B}^3$ does not contain any free boundary minimal Möbius bands. Moreover, $\mathbb {B}^3$ is not known to contain infinite families of pairwise noncongruent, embedded free boundary minimal surfaces all of which have the same topology, even though *k*-tuples of surfaces with these properties have been discovered in [4, 21] for every $k\in \mathbb {N}$. Allowing for codimension 2, Fraser and Schoen [15] characterised the *critical Möbius band*, a free boundary minimal surface in $\mathbb {B}^4$. Based on this work, Fraser and Sargent [13] classified $\mathbb {S}^1$-invariant free boundary minimal annuli and Möbius bands in $\mathbb {B}^m$ for m≥4.

The free boundary problem solved in Theorem 1.1 can also be compared with the Dirichlet problem in toroids: Area minimising Möbius bands with prescribed boundary on a mean convex torus have been found in [20, Corollary 6.3]. Complete minimal Möbius bands immersed in $\mathbb {R}^3$ have been studied in [30, 31, 33, 35].

We prove Theorem 1.1 in Section 5 using an equivariant, 1-parameter min-max scheme. Previous constructions of this type relied on area estimates which are uniform with respect to the order of the symmetry group [3, 12, 23]. In this context, property (iii) stating that the area of $\mathcal {O}_n$ grows linearly with *n* is a remarkable novelty. Area estimates are crucial for the min-max construction: The strict lower area bound is related with the width estimate established in Section 3. The strict upper area bound relies on the assumption $n\ge 5(\rho +1)/2$ and ensures that $\mathcal {O}_n$ is not just a $\mathbb {D}_n$-equivariant union of 2*n* planar discs, as detailed in Sections 2 and 5. We prove these estimates by employing a combination of new ideas tailored to the toroidal setting. Another key ingredient is our characterisation of all free boundary minimal discs in $M^{\rho }$, which is presented in Section 4.

## Construction of helicoidal sweepouts

The goal of this section is to construct an effective 1-parameter $\mathbb {D}_n$-sweepout of $M^{\rho }$, that is a family $\{\Sigma _t\}_{t\in [0,1]}$ of $\mathbb {D}_n$-equivariant subsets of $M^{\rho }$ such that $\Sigma _t$ is a smooth, properly embedded surface in $M^{\rho }$ for every t∈]0,1[, varying smoothly in t∈]0,1[ and continuously, in the sense of varifolds, for t∈[0,1] (cf. [12, Definition 1.1]). The novel idea is to use helicoids to construct suitable sweepout slices in cylinders and then map them to $M^{\rho }$.

Cylinders and translations. We denote the Cartesian coordinates on $\mathbb {R}^3$ by $x_1,x_2,x_3$ and the corresponding standard orthonormal basis by $e_1,e_2,e_3$. Let $\mathbb {B}^2=\{(x_1,x_2)\in \mathbb {R}^2\,{:}\, x_1^2+x_2^2\le 1\}$ denote the closed Euclidean unit disc. For the construction of surfaces in $M^{\rho }$ it is convenient to consider the Euclidean unit cylinder $Z:=\mathbb {B}^2\times \mathbb {R}\subset \mathbb {R}^3$ and the covering map $\Phi _\ell :Z\rightarrow M^{\rho }$ defined for ℓ>0 by

3 $$\begin{aligned} \Phi _\ell (x_1,x_2,x_3)&=\begin{pmatrix} (\rho +x_1)\cos (2x_3/\ell )\\ (\rho +x_1)\sin (2x_3/\ell ) \\ x_2 \end{pmatrix}. \end{aligned}$$

For any $\alpha \in [0,1]$ and any ℓ>0 we introduce the notation

4 $$\begin{aligned} Z_{\ell \alpha }&:=\mathbb {B}^2\times [0,\ell \pi \alpha ],&M^{\rho }_\alpha :=\Phi _\ell (Z_{\ell \alpha }). \end{aligned}$$

$M^{\rho }_0$ is a vertical slice through $M^{\rho }$ at toroidal angle 0. For 0<α<1 the set $M^{\rho }_\alpha$ is a torus sector with toroidal angle $2\pi \alpha$ and the restriction of $\Phi _\ell$ to $Z_{\ell \alpha }$ is a diffeomorphism onto its image. For α=1 the restriction of $\Phi _\ell$ to $Z_\ell =\mathbb {B}^2\times [0,\ell \pi ]$ is surjective and we simply have $M^{\rho }_1=M^{\rho }$. The following lemma facilitates area estimates for surfaces in $M^{\rho }$. Here and in the following, $\mathscr {H}^2$ denotes the 2-dimensional Hausdorff measure such that $\mathscr {H}^2(\Sigma )$ is the area of a surface $\Sigma \subset \mathbb {R}^3$.

### Lemma 2.1

Let $\Phi _\ell :Z_\ell \rightarrow M^{\rho }$ be as in (3) and $\Sigma \subset Z_\ell$ any properly embedded surface. Then

$$ \frac{2(\rho -1)}{\ell }\mathscr {H}^2(\Sigma ) \le \mathscr {H}^2\bigl (\Phi _\ell (\Sigma )\bigr ) \le \frac{2(\rho +1)}{\ell }\mathscr {H}^2(\Sigma ). $$

### Proof

Let $D\Phi _\ell$ be the Jacobian matrix of $\Phi _\ell$. Then $(D\Phi _\ell )^{\intercal }\cdot (D\Phi _\ell )$ is a diagonal matrix with nonzero entries $1,1,a^2$ for $a=2(\rho +x_1)/\ell$. In particular, $|\det (D\Phi _\ell )|=|a|$. Since $|x_1|\le 1$ the claim follows. □

Let $\textsf {T}_{y}(x)=x+y$ denote the translation by *y* in $\mathbb {R}^3$. Given $n\in \mathbb {N}$ let $G_n$ be the subgroup of Euclidean isometries acting on $Z=\mathbb {B}^2\times \mathbb {R}$ generated by the rotation $\textsf {R}_{e_1}^{\pi }$ and the vertical translation $\textsf {T}_{(\ell \pi /n)e_3}$. Let $\langle \textsf {T}_{\ell \pi e_3}\rangle$ denote the subgroup generated by $\textsf {T}_{\ell \pi e_3}$. The quotient

5 $$\begin{aligned} \tilde{Z}_{\ell }&:=Z/\langle \textsf {T}_{\ell \pi e_3}\rangle \end{aligned}$$

is a solid torus with mean convex boundary. In analogy to (2) we define for each $k\in \mathbb {Z}$

6 $$\begin{aligned} \zeta _k:=\Bigl \{\Bigl (r,0,\frac{\ell \pi k}{2n}\Bigr )\,{:}\, r\in [-1,1]\Bigr \}\subset Z. \end{aligned}$$

We also introduce the notation $\tilde{\zeta }_k\subset \tilde{Z}_{\ell }$ for the image of $\zeta _k$ under the quotient map $\varpi :Z\rightarrow \tilde{Z}_\ell$. Definitions (3), (2) and (6) are compatible in the following sense.

### Lemma 2.2

The map $\Phi _\ell :Z\rightarrow M^{\rho }$ defined in (3) descends to the quotient $\tilde{Z}_{\ell }$, where it becomes a $\mathbb {D}_n$-equivariant diffeomorphism for any $n\in \mathbb {N}$. Moreover, $\Phi _\ell (\zeta _k)=\xi _k$ for every *k*.

### Proof

Since sine and cosine are 2π-periodic functions, it is clear that $\Phi _\ell \circ \textsf {T}_{\ell \pi e_3}=\Phi _\ell$. Recalling $\textsf {R}_{e_1}^{\pi }(x_1,x_2,x_3)=(x_1,-x_2,-x_3)$ it follows by definition that $\Phi _\ell \circ \textsf {R}_{e_1}^{\pi }=\textsf {R}_{e_1}^{\pi }\circ \Phi _\ell$. Moreover, $\Phi _\ell \circ \textsf {T}_{(\ell \pi /n) e_3} =\textsf {R}_{e_3}^{2\pi /n}\circ \Phi _\ell$ it is easy to verify. We conclude that the group action of the factor group $G_n/\langle \textsf {T}_{\ell \pi e_3}\rangle$ on $\tilde{Z}_{\ell }$ is isomorphic to the action of $\mathbb {D}_n$ on $M^{\rho }$. The statement $\Phi _\ell (\zeta _k)=\xi _k$ follows directly from (2) and (6). □

Free boundary helicoids. The helicoid $X:=\{(x_1,x_2,x_3)\in \mathbb {R}^3\,{:}\, x_1\tan x_3=x_2\}$ is a complete, embedded, singly-periodic, simply connected minimal surface (cf. [8, § 1.2.2]). Its minimality was shown by Jean Baptiste Meusnier [32] in 1776. The helicoid X is a ruled surface parametrised by $\mathbb {R}^2\ni (u,v)\mapsto (v\cos u,v\sin u,u)$. In particular, any rescaling ℓX for ℓ>0 of the helicoid intersects the boundary of the Euclidean unit cylinder $Z=\mathbb {B}^2\times \mathbb {R}$ orthogonally; thus $Z\cap \ell X$ is a free boundary minimal surface in *Z*. Recalling (6), we notice that $\zeta _k$ intersects $Z\cap \tfrac{\ell }{n}X$ orthogonally for every odd *k* and that $Z\cap \tfrac{\ell }{n}X\cap \{x_2=0\}$ coincides with the union of all $\zeta _k$ with even *k*. Moreover, $Z\cap \tfrac{\ell }{n}X$ is $G_n$-equivariant and descends to a $\mathbb {D}_n$-equivariant free boundary minimal surface in the quotient $\tilde{Z}_{\ell }$ defined in (5). The following lemma indicates that the area of a helicoid’s period inside a cylinder is decreasing under horizontal translation.

### Lemma 2.3

Given ℓ>0 let $Z_\ell =\mathbb {B}^2\times [0,\ell \pi ]$ and let $X_{\ell ,s}:=(Z_\ell +(s,0,0))\cap \ell X$ for $s\in \mathbb {R}$. Then $\mathscr {H}^2(X_{\ell ,s})=\mathscr {H}^2(X_{\ell ,-s})$ is strictly decreasing in s>0.

### Proof

**Fig. 2:**
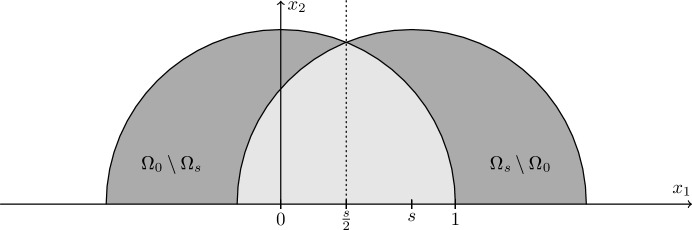
The upper half-disc $\Omega _0$ and its translation $\Omega _s=\Omega _0+(s,0)$

Given $s\in \mathbb {R}$ let $\Omega _s=\{x=(x_1,x_2)\in \mathbb {R}^2\,{:}\, (x_1-s)^2+x_2^2\le 1,~x_2>0\}$ be the laterally translated upper half-disc (see Fig. 2). Consider the function $w:\Omega _s\rightarrow ]0,\ell \pi [$ given by

7 $$\begin{aligned} w(x)&={\left\{ \begin{array}{ll} \ell \arctan (x_2/x_1) &  \text { if } x_1>0, \\ \ell \pi /2 &  \text { if } x_1=0, \\ \ell \arctan (x_2/x_1)+\ell \pi &  \text { if } x_1<0, \end{array}\right. } \end{aligned}$$

where $\arctan :\mathbb {R}\rightarrow ]-\frac{\pi }{2},\frac{\pi }{2}[$ is the inverse of $\tan :]-\frac{\pi }{2},\frac{\pi }{2}[\rightarrow \mathbb {R}$. By definition, the graph of *w* coincides with the surface $X_{\ell ,s}^{+}:=X_{\ell ,s}\cap \{x_2>0\}$ and by symmetry of X we have

8 $$\begin{aligned} \mathscr {H}^2(X_{\ell ,s})&=2\mathscr {H}^2(X_{\ell ,s}^+) =2\int _{\Omega _s}\sqrt{1+|\nabla w|^2}\,dx =2\int _{\Omega _s}\sqrt{1+\ell ^2|x|^{-2}}\,dx \end{aligned}$$

for every $s\in \mathbb {R}$. Let $\textsf {F}_0:(x_1,x_2)\mapsto (-x_1,x_2)$ and let $f(x):=\sqrt{1+\ell ^2|x|^{-2}}$. Then $f\circ \textsf {F}_0=f$ and $\textsf {F}_0(\Omega _s)=\Omega _{-s}$. From (8) we then obtain $\mathscr {H}^2(X_{\ell ,s})=\mathscr {H}^2(X_{\ell ,-s})$ as claimed.

Let us now assume s>0 and consider the reflection $\textsf {F}_s:(x_1,x_2)\mapsto (s-x_1,x_2)$ in $\mathbb {R}^2$ across $\{x_1=s/2\}$. Then $\Omega _s\setminus \Omega _0=\textsf {F}_s(\Omega _0\setminus \Omega _s)$ as visualised in Fig. 2. Since $f\in L_{\text {loc}}^1(\mathbb {R}^2)$, we have

9 $$\begin{aligned} \int _{\Omega _s}f\,dx -\int _{\Omega _0}f\,dx&=\int _{\Omega _s\setminus \Omega _0}f\,dx -\int _{\Omega _0\setminus \Omega _s}f\,dx =\int _{\Omega _0\setminus \Omega _s}(f\circ \textsf {F}_s)-f\,dx. \end{aligned}$$

Notice that $x_1<s/2$ and thus $(s-x_1)^2>x_1^2$ for every $x=(x_1,x_2)\in \Omega _0\setminus \Omega _s$. Hence, we directly obtain $f\bigl (\textsf {F}_s(x)\bigr )<f(x)$ for every $x\in \Omega _0\setminus \Omega _s$. Moreover, for every fixed $x\in \Omega _0\setminus \Omega _s$,

$$\begin{aligned} \frac{\partial }{\partial s}f\bigl (\textsf {F}_s(x)\bigr )&=\frac{\partial }{\partial s}\sqrt{1+\frac{\ell ^2}{(s-x_1)^2+x_2^2}} =\frac{-(s-x_1)\ell ^2}{\bigl ((s-x_1)^2+x_2^2\bigr )^2f\bigl (\textsf {F}_s(x)\bigr )}<0. \end{aligned}$$

Since additionally, $\Omega _0\setminus \Omega _{s_1}\subset \Omega _0\setminus \Omega _{s_2}$ for every $0<s_1<s_2$ we conclude that quantity (9) is strictly decreasing in s>0 and the claim follows. □

As a direct consequence of Lemma 2.3, we obtain a proof of the following Morse index estimate. While not required for the remainder of the article, this result is of independent interest being an example of index growth not imputable to topology (cf. [5] for higher-dimensional scenarios).

### Corollary 2.4

Let $\tilde{Z}_{\ell }$ be as in (5) and let $\tilde{X}_n$ be the $\mathbb {D}_n$-equivariant free boundary minimal surface in $\tilde{Z}_{\ell }$ which lifts to $Z\cap \frac{\ell }{n}X$ in *Z*. Then the Morse index of $\tilde{X}_n$ is at least *n*.

### Proof

The surface $\tilde{X}_n$ can be divided into *n* pairwise isometric domains $\Omega _1,\ldots ,\Omega _n$ by removing the segments $\tilde{\zeta }_k$ for all even *k*, recalling (6). By [6, Corollary 3.2 (i)] the Morse index of $\tilde{X}_n$ is bounded from below by *n* times the index of the Jacobi operator *J* on $\Omega _1$ subject to the Dirichlet boundary condition on $\tilde{\zeta }_0\cup \tilde{\zeta }_2$. We apply Lemma 2.3 with ℓ/n in place of ℓ to obtain a variation $\Omega _1(s)$ of $\Omega _1$ defined for some $-\varepsilon<s<\varepsilon$, with (strictly) maximal area at s=0. The corresponding variation vector field at s=0 vanishes on the segments $\tilde{\zeta }_0$ and $\tilde{\zeta }_2$ and therefore induces a negative direction for *J* with the aforementioned boundary condition. □

### Remark 2.5

Let *M* be any compact, three-dimensional ambient manifold with nonnegative Ricci curvature and *convex* boundary. Lima [26, Theorem 4] proved that the Morse index of any free boundary minimal surface Σ in *M* is bounded by a linear function of the sum of the genus and the number of boundary components of Σ. Corollary 2.4 shows that strict convexity of ∂M is a necessary assumption for this result because for any $n\in \mathbb {N}$ the free boundary minimal surface $\tilde{X}_{2n}$ in $\tilde{Z}_{\ell }$ has the topology of an annulus.

### Lemma 2.6

(Area of a piece of a helicoid) Let $X_{\ell ,0}$ be as in Lemma 2.3. Then

$$\begin{aligned} \mathscr {H}^2(X_{\ell ,0})&=\pi \sqrt{1+\ell ^2}+\pi \ell ^2\log \Bigl (\ell ^{-1}+\sqrt{\ell ^{-2}+1}\Bigr ). \end{aligned}$$

Moreover, $$\mathscr {H}^2(X_{\ell ,0})<2\pi $$ if $$\ell \le \frac{4}{5}$$.

### Proof

We recall that $X_{\ell ,0}$ is parametrised by $\phi (u,v)=(v\cos u,v\sin u,\ell u)$ defined on $[0,\pi [\times [-1,1]$. In particular, $X_{\ell ,0}$ intersects the positive level sets of the function

$$ \varrho (x_1,x_2,x_3):=\sqrt{x_1^2+x_2^2} $$

orthogonally. Hence, $|\nabla ^{X_{\ell ,0}}\varrho |=|\nabla \varrho |=1$, where $\nabla ^{X_{\ell ,0}}\varrho$ denotes the tangential gradient of the function ϱ restricted to $X_{\ell ,0}$. For every 0<r≤1 the set $X_{\ell ,0}\cap \varrho ^{-1}(r)$ is a double helix parametrised by $[0,\pi [\ni u\mapsto \phi (u,\pm r)$. Its total length is equal to

$$\begin{aligned} \mathscr {H}^1\bigl (X_{\ell ,0}\cap \varrho ^{-1}(r)\bigr )&=2\pi \sqrt{r^2+\ell ^2}. \end{aligned}$$

The function $r\mapsto 2\sqrt{r^2+\ell ^2}$ has a primitive given by $r\sqrt{r^2+\ell ^2}+\ell ^2\log \bigl (r+\sqrt{r^2+\ell ^2}\bigr )$. The coarea formula then implies

10 $$\begin{aligned} \mathscr {H}^2(X_{\ell ,0})&=\int ^{1}_{0}\biggl (\int _{X_{\ell ,0}\cap \varrho ^{-1}(r)}\frac{1}{|\nabla ^{X_{\ell ,0}}\varrho |}d\mathscr {H}^1\biggr )\,dr =\pi \int ^{1}_{0}2\sqrt{r^2+\ell ^2}\,dr \\\nonumber&=\pi \sqrt{1+\ell ^2}+\pi \ell ^2\log \Bigl (\ell ^{-1}+\sqrt{\ell ^{-2}+1}\Bigr ). \end{aligned}$$

The area (10) is clearly increasing in ℓ. For $\ell =\frac{4}{5}$ we evaluate $\mathscr {H}^2(X_{\frac{4}{5},0})\approx 1.95\pi <2\pi$. □

### Remark 2.7

An alternative approach is to compute the area of $X_{\ell ,0}$ as in equation (8). Indeed, integrating $\sqrt{1+\ell ^2|x|^{-2}}$ in polar coordinates over the unit disc yields the same integral as in equation (10). Numerically, we can solve the equation $\mathscr {H}^2(X_{\ell ,0})=2\pi$ for $\ell \approx 0.829$.

### Lemma 2.8

(Optimal sweepout for the helicoid) Given any ℓ>0, let $Z_\ell$ and $X_{\ell ,s}$ be as in Lemma 2.3 and let $\zeta _0,\zeta _1,\zeta _2\subset Z_\ell$ be as in (6) for n=1. There exists a sweepout $\{\Gamma _t\}_{t\in [0,1]}$ of $Z_{\ell }$ with the following properties.

(i) $\mathscr {H}^2(\Gamma _0)=\mathscr {H}^2(\Gamma _1)=\pi$ and $\mathscr {H}^2(\Gamma _t)\le \mathscr {H}^2(X_{\ell ,0})$ for every 0<t<1.
(ii) $\Gamma _t$ contains the segments $\zeta _{0}$ and $\zeta _{2}$, intersects $\zeta _1$ orthogonally, and is equivariant with respect to rotation by angle π around $\zeta _1$ for every 0<t<1.
(iii) $\Gamma _t$ descends to a smooth, properly embedded, $\mathbb {D}_1$-equivariant Möbius band $\tilde{\Gamma }_t$ in the quotient $\tilde{Z}_{\ell }$ defined in (5) for every 0<t<1.
(iv) For every 0<t<1 there exists a set $F_t\subset Z_\ell$ of finite perimeter such that
  $\Gamma _t$ is the relative perimeter of $F_t$ in the sense that $\Gamma _t\setminus \partial Z_\ell =\partial F_t\setminus \partial Z_\ell$;
  $\mathscr {H}^3(F_t)\rightarrow 0$ as t→0 and $\mathscr {H}^3(F_t)\rightarrow \mathscr {H}^3(Z_\ell )$ as t→1;
  $$t\rightarrow t_0$$ implies $$\mathscr {H}^3(F_t\mathbin {\triangle }F_{t_0})\rightarrow 0$$, where $$F_t\mathbin {\triangle }F_{t_0}:=(F_{t}\setminus F_{t_0})\cup (F_{t_0}\setminus F_{t})$$.

**Fig. 3:**
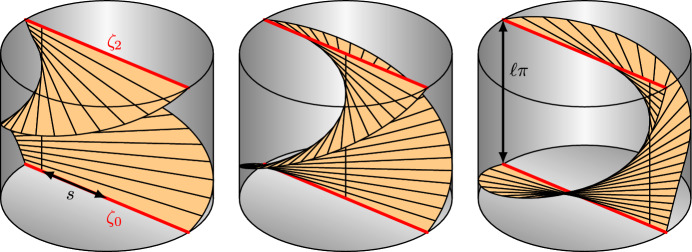
Horizontally translated helicoids $X_{\ell ,s}$ inside the cylinder $Z_\ell$

### Proof

**Fig. 4:**
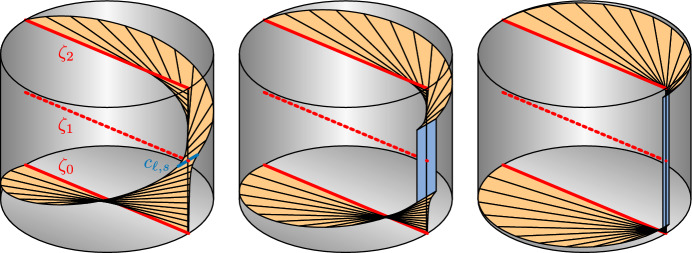
The surfaces $Y_{\lambda ,s}$ defined in (11): deforming a translated helicoid into two half-discs

Given -1<s<1, let $X_{\ell ,s}\subset Z_\ell$ be the horizontally translated helicoid introduced in Lemma 2.3 (see Fig. 3). By construction, $X_{\ell ,s}$ contains the segment $\zeta _0$. Moreover, the set $c_{\ell ,s}:=X_{\ell ,s}\cap \{x_3=\ell \pi /2\}$ is a straight segment of length δ(s) which intersects $\zeta _1$ orthogonally (see Fig. 4, left image). It divides $X_{\ell ,s}$ into two connected components which are congruent via rotation $\textsf {R}_{\zeta _1}^{\pi }$ by angle π around $\zeta _1$. Let $\Lambda _s$ be the connected component of $X_{\ell ,s}\setminus c_{\ell ,s}$ containing $\zeta _0$. Given $0<\lambda \le 1$, consider the map $f_\lambda :(x_1,x_2,x_3)\mapsto (x_1,x_2,\lambda x_3)$ and the union

11 $$\begin{aligned} Y_{\lambda ,s}:=f_\lambda (\Lambda _s)\cup I_{\lambda ,s} \cup \textsf {R}_{\zeta _1}^{\pi }f_\lambda (\Lambda _s), \end{aligned}$$

where $I_{\lambda ,s}$ is the planar, rectangular strip of vertical length $(1-\lambda )\ell \pi$ and horizontal width $\delta (s)=2\sqrt{1-s^2}$ connecting $f_\lambda (\Lambda _s)$ and $\textsf {R}_{\zeta _1}^{\pi }f_\lambda (\Lambda _s)$ as visualised in Fig. 4. Since vertical scaling $f_\lambda$ by $0<\lambda \le 1$ does not increase the area of $\Lambda _s$, we have

12 $$\begin{aligned} \mathscr {H}^2(Y_{\lambda ,s})&=2\mathscr {H}^2\bigl (f_\lambda (\Lambda _s)\bigr )+\mathscr {H}^2(I_{\lambda ,s}) <\mathscr {H}^2(X_{\ell ,s})+2\ell \pi \sqrt{1-s^2} \end{aligned}$$

for all $0<\lambda \le 1$. We may regularise $Y_{\lambda ,s}$ to obtain a smooth, $\textsf {R}_{\zeta _1}^{\pi }$-equivariant, properly embedded surface in $Z_\ell$ which depends smoothly on λ,s and still satisfies inequality (12). (We do not relabel the regularised surface.) By Lemma 2.3 applied to the right hand side in (12), there exists $s_0\in ]0,1[$ such that $\mathscr {H}^2(Y_{\lambda ,s})<\mathscr {H}^2(X_{\ell ,0})$ for all $s\in ]-1,-s_0]\cup [s_0,1[$ and all $0<\lambda \le 1$. Let now $\lambda :[s_0,1[\rightarrow ]0,1]$ be a smooth, decreasing function of *s* such that $\lambda (s_0)=1$ and $\lambda (s)\rightarrow 0$ as s→1. For any 0<t<1 we define

13 $$\begin{aligned} \Gamma _t:={\left\{ \begin{array}{ll} X_{\ell ,2t-1} &  \text { if }|2t-1|<s_0, \\ Y_{\lambda (|2t-1|),\,2t-1} &  \text { if }|2t-1|\ge s_0. \end{array}\right. } \end{aligned}$$

Lemma 2.3 and the choice of $s_0$ imply $\mathscr {H}^2(\Gamma _t)\le \mathscr {H}^2(X_{\ell ,0})$ for all 0<t<1. From its explicit parametrisation as a ruled surface or as a graph almost everywhere over $\mathbb {B}^2$ as in (7), it is evident that $\Gamma _t$ converges (in the sense of varifolds) to the union of two horizontal half-discs as t→0 respectively t→1 (see Fig. 4, right image). Defining $\Gamma _0$ and $\Gamma _1$ as the respective limit, we obtain (i). Moreover, $\Gamma _t$ inherits properties (ii) and (iii) from $X_{\ell ,s}$. Finally, we define $F_t$ as the connected component of $Z_\ell \setminus \Gamma _t$ containing the end point $(-1,0,\ell \pi /2)$ of $\zeta _1$ to prove (iv). □

### Corollary 2.9

(Sweepout construction) For every ρ>2 and every integer $n\ge 5(\rho +1)/2$ there exists a $\mathbb {D}_n$-sweepout $\{\Sigma _t\}_{t\in [0,1]}$ of $M^{\rho }$ with the following properties.

(i) $\mathscr {H}^2(\Sigma _0)=\mathscr {H}^2(\Sigma _1)=n\pi$ and $\mathscr {H}^2(\Sigma _t)<2n\pi$ for every t∈[0,1].
(ii) For every t∈]0,1[ the surface $\Sigma _t$ contains the segment $\xi _k$ defined in (2) if *k* is even and intersects it orthogonally if *k* is odd.
(iii) For every t∈]0,1[ the surface $\Sigma _t$ is a Möbius band if *n* is odd, or an annulus if *n* is even.
(iv) For every t∈[0,1] there exists a set $E_t\subset M^{\rho }_{1/n}$ of finite perimeter such that
  $\Sigma _t\cap M^{\rho }_{1/n}$ is the relative perimeter of $E_t$ in $M^{\rho }_{1/n}$ for every 0<t<1;
  $$E_0=\emptyset $$ and $$E_1=M^{\rho }_{1/n}$$;
  $$\displaystyle \lim _{t\rightarrow t_0}\mathscr {H}^3(E_t\mathbin {\triangle }E_{t_0})=0$$ for any $$t_0\in [0,1]$$.

### Proof

Let the real number ρ>2 and the integer $n\ge 5(\rho +1)/2$ be fixed. Choosing $\ell =2(\rho +1)$, let $\{\Gamma _t\}_{t\in [0,1]}$ be the sweepout of $Z_{\ell /n}$ constructed in Lemma 2.8 (which we apply with ℓ/n in place of ℓ). By assumption $\ell /n\le 4/5$, therefore Lemma 2.8 (i) and Lemma 2.6 imply $\mathscr {H}^2(\Gamma _t)<2\pi$ for every t∈[0,1]. Recalling the surjective map $\Phi _\ell :Z_\ell \rightarrow M^{\rho }$ from (3), we define for every t∈[0,1]

14 $$\begin{aligned} \hat{\Gamma }_t&:=\bigcup _{k=0}^{n-1}\textsf {T}_{(\ell \pi k/n)e_3}(\Gamma _t),&\Sigma _t&:=\Phi _\ell (\hat{\Gamma }_t). \end{aligned}$$

Lemma 2.1 and our choice of ℓ imply $\mathscr {H}^2(\Sigma _t)\le \mathscr {H}^2(\hat{\Gamma }_t)=n\mathscr {H}^2(\Gamma _t)<2n\pi$. By construction, $\Sigma _0$ and $\Sigma _1$ coincide with the union

$$ \bigcup _{k=0}^{n-1}\textsf {R}^{2\pi k/n}_{e_3}M^{\rho }_0 $$

of *n* vertical discs in $M^{\rho }$. This completes the proof of claim (i). Claim (ii) follows directly from Lemma 2.8 (ii) and Lemma 2.2, which also imply that $\Sigma _t$ is a properly embedded, $\mathbb {D}_n$-equivariant surface in $M^{\rho }$. The boundary $\partial \Gamma _t\cap \partial Z$ is a half-twist of a double-helix (see Figs. 3–4). Therefore, $\Sigma _t$ comprises *n* such half-twists and claim (iii) follows. Recalling the finite perimeter set $F_t\subset Z_{\ell /n}$ from Lemma 2.8 (iv) we define $E_t:=\Phi _\ell (F_t)$. Then claim (iv) follows directly from Lemma 2.8 (iv) and the fact that $\Phi _\ell$ restricts to a diffeomorphism $Z_{\ell /n}\rightarrow M^{\rho }_{1/n}$. □

## Width estimate

Let $\{\Sigma _t\}_{t\in [0,1]}$ be the $\mathbb {D}_n$-sweepout of $M^{\rho }$ constructed in Corollary 2.9. Its $\mathbb {D}_n$-saturation Π is defined as the set of all $\{f(t,\Sigma _t)\}_{t\in [0,1]}$, where $f:[0,1]\times M^{\rho }\rightarrow M^{\rho }$ is smooth such that f(t,·) is a diffeomorphism which commutes with the $\mathbb {D}_n$-action for all t∈[0,1] and coincides with the identity for $t\in \{0,1\}$ (cf. [12, Definition 1.3]). The corresponding min-max width of Π is

$$ W_\Pi :=\inf _{\{\Lambda _t\}\in \Pi ~}\sup _{t\in [0,1]}\mathscr {H}^2({\Lambda _t}). $$

The width estimate $W_\Pi >\max \{\mathscr {H}^2(\Sigma _0),\mathscr {H}^2(\Sigma _1)\}$ is the essential condition for applying the min-max theorem (cf. [12, Theorem 1.4] and references therein). The proof of the width estimate typically relies on the (relative) isoperimetric inequality in the ambient space. Indeed, property (iv) in Corollary 2.9 ensures that the sweepout in question is nontrivial and this is what allows for the comparison with isoperimetric bounds.

### Lemma 3.1

(Isoperimetric inequality in the unit ball [2]) Any surface Σ dividing the Euclidean unit ball $\mathbb {B}^3\subset \mathbb {R}^3$ into two equal volumes has area $\mathscr {H}^2(\Sigma )\ge \pi$.

### Lemma 3.2

(Isoperimetric inequality in cylinders) Given ℓ>0, let $F\subset Z_\ell =\mathbb {B}^2\times [0,\ell \pi ]$ be any set with finite perimeter and Lebesgue measure $\mathscr {H}^3(F)=\frac{1}{2}\mathscr {H}^3(Z_\ell )$. Then its relative perimeter satisfies $P(F;Z_\ell )\ge \min \{\pi ,2\pi \ell \}$.

### Proof

Theorem 23 (d) in [38] implies that among all hypersurfaces in $Z_\ell$ dividing it in two equal volumes, the area is minimised either by $\mathbb {B}^2\times \{\ell \pi /2\}$ or by $[-1,1]\times [0,\ell \pi ]$. □

If $\ell <\frac{1}{2}$ then Lemma 3.2 is insufficient to establish the width estimate for the sweepout $\{\Gamma _t\}_{t\in [0,1]}$ constructed in Lemma 2.8, because $2\pi \ell <\pi =\mathscr {H}^2(\Gamma _0)$. The following crucial insight allows us to overcome this limitation of the isoperimetric inequality in cylinders.

### Lemma 3.3

Given ℓ>0 let $\varpi :Z\rightarrow \tilde{Z}_\ell$ be the quotient map associated with (5). Let $\tilde{\Sigma }\subset \tilde{Z}_\ell$ be any properly embedded Möbius band which allows an ambient diffeomorphism $\varphi :\tilde{Z}_\ell \rightarrow \tilde{Z}_\ell$ such that $\varphi (\tilde{\Sigma })=\varpi (Z\cap \frac{\ell }{n}X)$ for some $n\in \mathbb {Z}\setminus \{0\}$, where *X* denotes the helicoid as in Section 2. Then the projection map $p:\tilde{\Sigma }\rightarrow \mathbb {B}^2$ given by $(x_1,x_2,x_3)\mapsto (x_1,x_2)$ is surjective.

### Proof

Towards a contradiction, suppose that there exists $y\in \mathbb {B}^2\setminus p(\tilde{\Sigma })$. Then $\tilde{\gamma }:=\varpi (\{y\}\times \mathbb {R})$ is a simple closed curve in $\tilde{Z}_\ell \setminus \tilde{\Sigma }$. Let $h\in \mathbb {R}$ such that the disc $\tilde{D}:=\varpi (\mathbb {B}^2\times \{h\})\subset \tilde{Z}_\ell$ intersects the curve $\varphi (\tilde{\gamma })$ transversally. On the one hand, the curve $\varphi (\tilde{\gamma })$ has *odd* intersection number with $\tilde{D}$ because by definition the homotopy class of $\tilde{\gamma }$ generates the fundamental group of $\tilde{Z}_\ell$. On the other hand, since $\varphi (\tilde{\Sigma })$ is a helicoidal Möbius band by assumption, $\tilde{Z}_\ell \setminus \varphi (\tilde{\Sigma })$ is connected with the topology of a solid torus and $\tilde{D}\setminus \varphi (\tilde{\Sigma })$ has exactly two connected components (two half-discs, separated by a line segment) which divide $\tilde{Z}_\ell \setminus \varphi (\tilde{\Sigma })$ into exactly *two* connected components. Therefore, any closed curve in $\tilde{Z}_\ell \setminus \varphi (\tilde{\Sigma })$ which intersects the separating interface $\tilde{D}\setminus \varphi (\tilde{\Sigma })$ transversally, such as $\varphi (\tilde{\gamma })$, must intersect it an even number of times. This contradiction proves the surjectivity of *p*. □

### Lemma 3.4

(Width estimate) Let Π be the $\mathbb {D}_n$-saturation of the $\mathbb {D}_n$-sweepout $\{\Sigma _t\}_{t\in [0,1]}$ of $M^{\rho }$ constructed in Corollary 2.9 for ρ>2 and $n\ge 5(\rho +1)/2$. Then its min-max width satisfies

$$\begin{aligned} n\pi<W_\Pi <2n\pi . \end{aligned}$$

### Proof

The upper bound follows directly from Corollary 2.9 (i). For the lower bound, let $\{\Lambda _t\}_{t\in [0,1]}\in \Pi$ be arbitrary. By definition, there exists a smooth function $f:[0,1]\times M^{\rho }\rightarrow M^{\rho }$, where f(t,·) is a $\mathbb {D}_n$-equivariant diffeomorphism for all t∈[0,1] which coincides with the identity for $t\in \{0,1\}$, such that $\Lambda _t=f(t,\Sigma _t)$.

Corollary 2.9 (ii) and the properties of f(t,·) imply that $\xi _k\subset \Lambda _t$ for every even *k*, where we recall the segments $\xi _k$ from (2). By Corollary 2.9 (iv) there exists a continuous family $\{E_t\}_{t\in [0,1]}$ of finite perimeter sets in the torus sector $M^{\rho }_{1/n}$ such that $\Sigma _t\cap M^{\rho }_{1/n}$ is the relative perimeter of the set $E_t\subset M^{\rho }_{1/n}$ for all 0<t<1. We also define $E_t':=\textsf {R}_{e_1}^{\pi }(M^{\rho }_{1/n}\setminus E_t)$. Let $\Phi _\ell :Z\rightarrow M^{\rho }$ be as defined in (3). In the proof of Corollary 2.9 we chose the value 2(ρ+1) for ℓ in order to obtain upper area bounds using Lemma 2.1. Now, aiming for lower area bounds, we instead choose $\ell =2(\rho -1)$ and define for any 0<t<1

15 $$\begin{aligned} L_t&:=\Phi _\ell ^{-1}(\Lambda _t),&F_t&:=\Phi _\ell ^{-1}\bigl (f(t,E_t)\bigr ),&F_t'&:=\Phi _\ell ^{-1}\bigl (f(t,E_t')\bigr ). \end{aligned}$$

Then $L_t$ is a properly embedded, $\ell \pi /n$-periodic surface in $Z=\mathbb {B}^2\times \mathbb {R}$ containing $\zeta _k$ defined in (6) for every even *k* by Lemma 2.2. In particular, it passes to a properly embedded surface $\tilde{L}_t$ in the quotient $\tilde{Z}_{\ell /n}$ as defined in (5). Up to a vertical translation (which does not change area) we may assume that $L_t$ intersects $Z_\ell$ transversally. Lemma 2.1 and our choice of ℓ then imply

16 $$\begin{aligned} n\mathscr {H}^2(\tilde{L}_t) =\mathscr {H}^2(L_t\cap Z_\ell )&\le \mathscr {H}^2\bigl (\Phi _\ell (L_t\cap Z_\ell )\bigr ) =\mathscr {H}^2(\Lambda _t). \end{aligned}$$

Similarly, $\Phi _\ell ^{-1}(\Sigma _t)$ passes to a properly embedded surface $\tilde{\Gamma }_t$ in $\tilde{Z}_{\ell /n}$. Moreover, $\tilde{L}_t$ and $\tilde{\Gamma }_t$ are related by an ambient diffeomorphism. Recalling the definition (14) of $\Sigma _t$ and Lemma 2.8 (iii) we know that $\tilde{\Gamma }_t$ and hence $\tilde{L}_t$ is a topological Möbius band satisfying the hypothesis of Lemma 3.3. Hence, the projection map $p:\tilde{L}_t\rightarrow \mathbb {B}^2$ given by $(x_1,x_2,x_3)\mapsto (x_1,x_2)$ is surjective. Given any r>0 to be chosen, let $T_r=\{x\in \tilde{Z}_{\ell /n}\,{:}\, x_1^2+x_2^2\le r^2\}$. Since *p* is surjective and does not increase area,

17 $$\begin{aligned} \mathscr {H}^2(\tilde{L}_t\setminus T_r)&\ge \mathscr {H}^2\bigl (p(\tilde{L}_t\setminus T_r)\bigr ) =(1-r^2)\pi . \end{aligned}$$

**Fig. 5:**
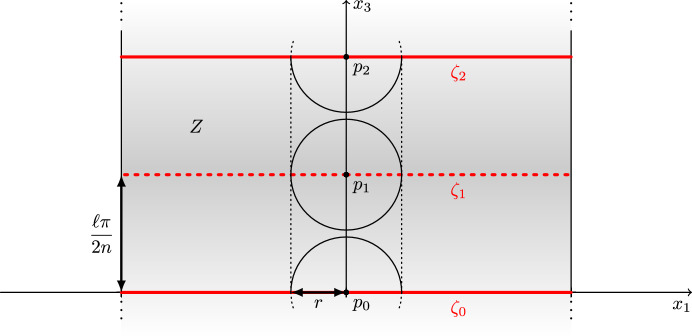
Vertical cross-section of the Euclidean unit cylinder *Z*

It remains to prove a refined estimate for $\mathscr {H}^2(\tilde{L}_t\cap T_r)$. Let $B_r(p_k)\subset Z$ denote the Euclidean ball of radius r>0 around the point $p_k=(0,0,k\ell \pi /(2n))\in \zeta _k$ for all $k\in \mathbb {Z}$. We may choose $r\in ]\ell \pi /(5n),\ell \pi /(4n)[$ such that $L_t$ intersects $\partial B_r(p_k)$ transversally and such that the balls $B_r(p_k)$ are disjoint for varying *k*, as shown in Fig. 5. We claim that there exists $t_0\in ]0,1[$ such that for every $k\in \mathbb {Z}$

18 $$\begin{aligned} \mathscr {H}^2\bigl (L_{t_0}\cap B_r(p_k)\bigr )\ge \pi r^2. \end{aligned}$$

If *k* is even, (18) follows from Lemma 3.1 (see also [39, Lemma 4.4]) and the fact that $L_{t_0}$ contains the segment $\zeta _k$ and is equivariant with respect to the rotation of angle π around $\zeta _k$ for any choice of $t_0\in ]0,1[$. Thus, it suffices to prove (18) for k=1, recalling the periodicity of $L_t$.

The surface $L_t$ divides the cylinder *Z* in two connected components: Let $\Omega _t$ denote the closure of the connected component of $Z\setminus L_t$ containing the interior of the set $F_t$ defined in (15). Then $\Omega _t$ is $2\ell \pi /n$-periodic and the closure of any connected component of $F_t\cup F_t'$ is a fundamental domain for $\Omega _t$. The intersection $Q_t:=\Omega _t\cap B_r(p_1)$ is a possibly empty set of finite perimeter. As stated in Corollary 2.9 (iv), $\{E_t\}_{t\in ]0,1[}$ and thus $\{E_t'\}_{t\in ]0,1[}$ are continuous families of sets of finite perimeter. This property is inherited by $F_t\cup F_t'$ and thus by $Q_t$, i. e.

19 $$\begin{aligned} \displaystyle \lim _{t\rightarrow t_0}\mathscr {H}^3(Q_t\mathbin {\triangle }Q_{t_0})=0 \end{aligned}$$

for any $t_0\in ]0,1[$. By definition, $\Phi _\ell (B_r(p_1))\subset M^{\rho }_{1/n}$. Since $E_0=\emptyset$ and $E_1=M^{\rho }_{1/n}$, and since f(t,·) is the identity for $t\in \{0,1\}$, we have $Q_0=\emptyset$ and $Q_1=B_r(p_1)$. By (19) there exists $t_0\in ]0,1[$ such that $\mathscr {H}^3(Q_{t_0})=\tfrac{1}{2}\mathscr {H}^3\bigl (B_r(p_1)\bigr )$. Claim (18) then follows from Lemma 3.1.

By choice of *r*, estimate (18) implies $\mathscr {H}^2(\tilde{L}_t\cap T_r)\ge 2\pi r^2$ (cf. Fig. 5). Combined with inequality (17) we obtain $\mathscr {H}^2(\tilde{L}_t)\ge (1+r^2)\pi$ and thus $\mathscr {H}^2(\Lambda _t)\ge n(1+r^2)\pi$ by (16). Since $\{\Lambda _t\}_{t\in [0,1]}\in \Pi$ is arbitrary, and since $r>\ell \pi /(5n)=2(\rho -1)\pi /(5n)$ we obtain

20 $$\begin{aligned} W_\Pi&>n\biggl (1+ \Bigl (\frac{2(\rho -1)\pi }{5n}\Bigr )^2\biggr )\pi \end{aligned}$$

implying $W_\Pi >n\pi$ as claimed. □

### Remark 3.5

The width estimate (20) being quantitative in terms of the parameter ρ and the order 2*n* of the symmetry group is a novelty compared with the respective arguments in [3, 23, 39]. Moreover, it is remarkable that the upper and lower bounds on the width *increase* with the order of the symmetry group rather than being uniform in *n*.

## Uniqueness of free boundary minimal discs in cylinders and toroids

Nitsche [34] proved that the planar, equatorial disc is unique in the class of immersed free boundary minimal discs in the Euclidean unit ball $\mathbb {B}^3$ up to ambient isometries. Fraser and Schoen [14] later extended this result to higher codimensions. In more general ambient manifolds, free boundary minimal discs are harder to characterise. In certain ellipsoids for example a surprising variety of nonplanar free boundary minimal discs have been discovered [18, 36, 39]. In contrast, a free boundary minimal disc in a cylinder is necessarily planar and orthogonal to the cylinder axis by Corollary 5 in [11, § 5.5]. This interlude section contains an alternative proof of this result in a more general setting. Based on this, we characterise all free boundary minimal discs in the toroid $M^{\rho }$, which is a key ingredient for the proof of Theorem 1.1.

### Theorem 4.1

Let $(\mathbb {B}^2,g_{\mathbb {B}^2})$ be the compact Euclidean unit disc in $\mathbb {R}^2$ and let $f:\mathbb {B}^2\rightarrow ]0,\infty [$ be smooth. Let Σ be any compact, connected free boundary minimal surface in the Riemannian manifold (*Z*, *g*), where $Z=\mathbb {B}^2\times \mathbb {R}$ is a cylinder equipped with Cartesian coordinates $(x_1,x_2,x_3)$ and $g=g_{\mathbb {B}^2}+f^2\,dx_3^2$. Then $\Sigma =\mathbb {B}^2\times \{h\}$ for some $h\in \mathbb {R}$.

### Proof

We may assume that Σ is the image of some conformal map $u=(u_1,u_2,u_3):\Omega \rightarrow Z$. Denoting the partial derivatives of *u* by $\partial _1 u$ and $\partial _2u$, we have $|\partial _1u|_g^2=|\partial _2u|_g^2$ and $g(\partial _1u,\partial _2u)=0$. Consider the diffeomorphism $\varphi _t:Z\rightarrow Z$ given by $\varphi _t(x_1,x_2,x_3)=(x_1,x_2,e^{t}x_3)$. By the area formula [27, Theorem 8.1]

$$\begin{aligned} \mathscr {H}^2\bigl (\varphi _t\bigl (\Sigma \bigr )\bigr )&=\int _{\Omega }\sqrt{ |D\varphi _t\partial _1u|_g^2 |D\varphi _t\partial _2u|_g^2 -g(D\varphi _t\partial _1u,D\varphi _t\partial _2u)^2 }. \end{aligned}$$

Since $$|D\varphi _t\partial _ju|_g^2 =|\partial _ju|_g^2+(e^{2t}-1)(f\partial _ju_3)^2$$ and

$$ g(D\varphi _t\partial _1u,D\varphi _t\partial _2u) =(e^{2t}-1)(f\partial _1u_3)(f\partial _2u_3), $$

and since *f* and thus *g* are independent of $x_3$, the stationarity of Σ implies that

$$\begin{aligned} 0=\frac{\partial }{\partial t}\Big \vert _{t=0}\mathscr {H}^2\bigl (\varphi _t\bigl (\Sigma \bigr )\bigr )&=\int _{\Omega } \frac{|\partial _1u|_g^2(f\partial _2u_3)^2+|\partial _2u|_g^2(f\partial _1u_3)^2}{|\partial _1u|_g|\partial _2u|_g} \\&=\int _{\Omega }\Bigl ((\partial _1u_3)^2+(\partial _2u_3)^2\Bigr )f^2. \end{aligned}$$

We conclude that $u_3$ is constant, relying on the assumption that the domain is connected. □

### Corollary 4.2

Any embedded free boundary minimal disc Σ in the toroid $M^{\rho }$ defined in (1) coincides with the vertical planar slice $M^{\rho }_0$ up to a rotation of the coordinate system.

### Proof

Let $\Phi _\ell :Z\rightarrow M^{\rho }$ be the covering map defined in (3) with ℓ=1 and let $\iota :\Sigma \hookrightarrow M^{\rho }$ be the inclusion map. Since the disc Σ is simply connected, the lifting criterion for covering spaces [19, Proposition 1.33] guarantees the existence of a continuous lift $\tilde{\iota }:\Sigma \rightarrow Z$ such that $\Phi _\ell \circ \tilde{\iota } = \iota$. Because Σ is compact and $\tilde{\iota }$ continuous, the image $\tilde{\iota }(\Sigma )$ is a compact, connected free boundary minimal surface in the Riemannian manifold $(Z,\Phi _\ell ^*g_{M^{\rho }})$. Since $\Phi _\ell ^*g_{M^{\rho }}=g_{\mathbb {B}^2}+f^2\,d x_3^2$ for $f=2(\rho +x_1)/\ell$ the claim follows from Theorem 4.1. □

## Topological control

In this section we prove Theorem 1.1 using $\mathbb {D}_n$-equivariant min-max methods employing the sweepout constructed in Section 2 and the width estimate obtained in Section 3. The control of the topology of the resulting limit surface relies on the author’s previous work with Franz, specifically [12, Theorem 1.8] stating that the first Betti number $\beta _1$ and the genus complexity $\mathfrak {g}$ defined in [12, Definition 1.6] are lower semicontinuous along min-max sequences. However, if Σ is an annulus or a Möbius band then $\beta _1(\Sigma )=1$ and $\mathfrak {g}(\Sigma )=0$ in both cases. To distinguish between them, additional information is required. The following lemma implies that one cannot obtain an annulus from a Möbius band through surgery. It involves the boundary complexity $\mathfrak {b}$ defined in [12, Definition 1.6] as the sum of the number of boundary components minus 1 over each connected component with boundary. For example, an arbitrary finite union $\Sigma _0$ of topological spheres, discs and Möbius bands satisfies $\mathfrak {b}(\Sigma _0)=0$ while an annulus $\Sigma _1$ satisfies $\mathfrak {b}(\Sigma _1)=1$. Note that in general, the boundary complexity can increase through surgery.

### Lemma 5.1

Given a smooth, compact, topological Möbius band Σ which is properly embedded in some three-dimensional ambient manifold *M*, let $\hat{\Sigma }$ be obtained from Σ through surgery in the sense of [12, Definition 3.1]. Then $\mathfrak {b}(\hat{\Sigma })=0$. In particular, $\hat{\Sigma }$ does not contain any annuli.

### Proof

Focusing on the boundary complexity, it suffices to consider a surface $\hat{\Sigma }$ which is obtained from Σ by cutting away a half-neck in the sense of [12, Definition 3.1 (b)]. In this case, the Euler characteristics of Σ and $\hat{\Sigma }$ are related by $\chi (\hat{\Sigma })=\chi (\Sigma )+1=1$. We denote by $\hat{c}_{\textrm{O}}$ respectively $\hat{c}_{\textrm{N}}$ the number of orientable respectively nonorientable connected components of $\hat{\Sigma }$ and by $\hat{c}_{\textrm{b}}$ the number of its boundary components. By [12, Lemma 3.4], the genus complexity of $\hat{\Sigma }$ necessarily vanishes and [12, Corollary A.2] then implies

21 $$\begin{aligned} 1=\chi (\hat{\Sigma })=2\hat{c}_{\textrm{O}}+\hat{c}_{\textrm{N}}-\hat{c}_{\textrm{b}}. \end{aligned}$$

Since $\partial \Sigma$ is connected we have either $\hat{c}_{\textrm{b}}=1$ or $\hat{c}_{\textrm{b}}=2$. In the first case, the surface stays connected, i. e. $\hat{c}_{\textrm{O}}+\hat{c}_{\textrm{N}}=1$, and (21) implies $\hat{c}_{\textrm{O}}=1$ and $\hat{c}_{\textrm{N}}=0$. Thus, $\hat{\Sigma }$ is a topological disc and any further surgery operation cannot increase the boundary complexity. In the second case, $2\hat{c}_{\textrm{O}}+\hat{c}_{\textrm{N}}=3$ and since necessarily $\hat{c}_{\textrm{N}}\le 2$ we have $\hat{c}_{\textrm{O}}=1=\hat{c}_{\textrm{N}}$. Since each of the two connected components must have boundary, $\hat{\Sigma }$ is the union of a disc and a Möbius band. The claim follows by iterating the argument. □

### Proof (Proof of Theorem 1.1)

Given ρ>2 and $n\ge 5(\rho +1)/2$ let $\{\Sigma _t\}_{t\in [0,1]}$ be the $\mathbb {D}_n$-equivariant sweepout of $M^{\rho }$ constructed in Corollary 2.9. The width estimate stated in Lemma 3.4 and the mean-convexity of $\partial M^{\rho }$ imply that the min-max theorem [12, Theorem 1.4] applies: There exists a min-max sequence $\{\Sigma ^j\}_{j\in \mathbb {N}}$ converging in the sense of varifolds to $\Gamma := \sum _{i=1}^k m_i\Gamma _i$ for some $k\in \mathbb {N}$, where the varifolds $\Gamma _1,\ldots ,\Gamma _k$ are induced by pairwise disjoint, connected, embedded free boundary minimal surfaces in $M^{\rho }$ and where the multiplicities $m_1,\ldots ,m_k$ are positive integers. Moreover, Γ is $\mathbb {D}_n$-equivariant. Recalling Lemma 3.4, we have

22 $$\begin{aligned} |\Gamma |:= \sum _{i=1}^k m_i\mathscr {H}^2(\Gamma _i)&=W_\Pi \in ]n\pi ,2n\pi [. \end{aligned}$$

Corollary 2.9 (iii) implies that all the surfaces in the min-max sequence have first Betti number $\beta _1(\Sigma ^j)=1$. Applying the topological lower semicontinuity result [12, Theorem 1.8], we obtain

23 $$\begin{aligned} \sum _{i=1}^k \beta _1(\Gamma _i)\le 1. \end{aligned}$$

Since $M^{\rho }\subset \mathbb {R}^3$ does not contain any closed minimal surfaces, (23) implies that each $\Gamma _i$ is either a topological disc, an annulus or a Möbius band, and that at most one of them is not a disc.

Towards a contradiction suppose that $\beta _1(\Gamma _i)=0$ for some $i\in \{1,\ldots ,k\}$. Up to relabelling we may assume i=1. Then $\Gamma _1$ is a free boundary minimal disc in $M^{\rho }$. The uniqueness result Corollary 4.2 implies that $\Gamma _1$ coincides with the vertical slice $M^{\rho }_0$ up to a rotation of the coordinate system. In particular, $\mathscr {H}^2(\Gamma _1)=\pi$. Being $\mathbb {D}_n$-equivariant, the union $\Gamma _1\cup \ldots \cup \Gamma _k$ also contains $V=\bigcup _{j=1}^n\textsf {R}^{2\pi j/n}_{e_3}\Gamma _1$ having area $\mathscr {H}^2(V)=n\pi$. If there exists $j\in \{1,\ldots ,k\}$ such that $\beta _1(\Gamma _j)=1$, then $\Gamma _j$ is disjoint from *V* which by $\mathbb {D}_n$-equivariance implies that the support of Γ contains at least *n* pairwise disjoint copies of $\Gamma _j$ contradicting (23).

We conclude that if $\Gamma _1$ is a disc then all $\Gamma _1,\ldots ,\Gamma _k$ are planar, vertical discs isometric to $M^{\rho }_0$. In this case, the $\mathbb {D}_n$-equivariance implies that |Γ| is an integer multiple of nπ contradicting (22). As a result we obtain k=1 and $\Gamma =m_1\Gamma _1$ with $\beta _1(\Gamma _1)=1$. In particular, the free boundary minimal surface $\mathcal {O}_n:=\Gamma _1$ is either an annulus or a Möbius band. It remains to prove properties (i)–(iii) stated in the theorem. We will also prove $m_1=1$ to obtain the area estimate stated in (iii).

(i) Let the integer $k\in \{0,\ldots ,2n-1\}$ be even. By construction, every surface along the min-max sequence contains the segment $\xi _k$ defined in (2). Consequently, $\xi _k\subset \mathcal {O}_n$ which also implies that the multiplicity $m_1$ is odd (see [23, § 7.3]). Now let the integer $k\in \{0,\ldots ,2n-1\}$ be odd. By [22, Lemma 3.4 (2)] (applied in a suitable ball in $M^{\rho }$ containing $\xi _k$) we have either $\xi _k\subset \mathcal {O}_n$ or $\mathcal {O}_n\cap \xi _k$ is finite (possibly empty) and every intersection is orthogonal. By Lemma 2.9 (ii), every surface along the min-max sequence intersects $\xi _k$ orthogonally. The equivariance with respect to rotation by angle π around $\xi _k$ then implies that if $\xi _k\subset \mathcal {O}_n$, the multiplicity $m_1\in \mathbb {N}$ is even by [23, Theorem 3.2.iv] (see also [22, Theorem 1.3.f]). This however contradicts the fact shown above that $m_1$ is odd. Hence, the intersection $\mathcal {O}_n\cap \xi _k$ is finite and orthogonal if not empty. It remains to prove that $\mathcal {O}_n\cap \xi _k$ is in fact nonempty. The quotient $\tilde{M^{\rho }}=M^{\rho }/\mathbb {Z}_n$ obtained by factoring out the cyclic subgroup of order *n* is a smooth, mean convex manifold. Let $\varpi :M^{\rho }\rightarrow \tilde{M^{\rho }}$ denote the quotient map. Since the surfaces $\mathcal {O}_n$ and $\Sigma ^j$ are $\mathbb {Z}_n$-equivariant in $M^{\rho }$, their quotients $\tilde{\mathcal {O}}_n=\varpi (\mathcal {O}_n)$ and $\tilde{\Sigma }^j=\varpi (\Sigma ^j)$ are smooth, properly embedded surfaces in $\tilde{M^{\rho }}$. Moreover, the sequence $\{\tilde{\Sigma }^j\}_{j\in \mathbb {N}}$ converges in the sense of varifolds to $m_1\tilde{\mathcal {O}}_n$ as $j\rightarrow \infty$ and is $\mathbb {Z}_2$-almost minimizing, where $\mathbb {Z}_2$ is the action of the group $\mathbb {D}_n$ reduced to the quotient $\tilde{M^{\rho }}$. Recalling Lemma 2.8 (iii) and Corollary 2.9, the surface $\tilde{\Sigma }^j$ has the topology of a Möbius band. We claim that $\tilde{\mathcal {O}}_n$ is also a Möbius band in $\tilde{M^{\rho }}$. A variant of the Riemann–Hurwitz formula (see e. g. [16, § IV.3]) implies $\beta _1(\tilde{\mathcal {O}}_n)=\beta _1(\mathcal {O}_n)=1$. Suppose that $\tilde{\mathcal {O}}_n$ is not a Möbius band, in which case it must be an annulus. Let $U\subset \tilde{M^{\rho }}$ be a thin tubular neighbourhood around $\tilde{\mathcal {O}}_n$. By [12, Theorem 4.11] we can apply a topological surgery procedure to all surfaces $\tilde{\Sigma }^j$ with sufficiently large *j*, resulting in $\mathbb {Z}_2$-equivariant surfaces $\hat{\Sigma }^j\subset U$ such that the sequence $\{\hat{\Sigma }^j\}_{j}$ still converges to $m_1\tilde{\mathcal {O}}_n$ in the sense of varifolds and such that the boundary complexity $\mathfrak {b}$ defined in [12, Definition 1.6] satisfies
  $$\begin{aligned} \mathfrak {b}(\tilde{\mathcal {O}}_n)&\le \liminf _{j\rightarrow \infty }\mathfrak {b}(\hat{\Sigma }^j). \end{aligned}$$
  If $\tilde{\mathcal {O}}_n$ is an annulus, then $\mathfrak {b}(\tilde{\mathcal {O}}_n)=1$ implying that $\mathfrak {b}(\hat{\Sigma }^j)\ge 1$ for all sufficiently large *j*. Using Lemma 5.1 we obtain a contradiction to the fact that $\hat{\Sigma }^j$ is obtained from the Möbius band $\tilde{\Sigma }^j$ via surgery. Consequently $\tilde{\mathcal {O}}_n$ is a topological Möbius band. Let $\tilde{\xi }_k=\varpi (\xi _k)$ for $k\in \{0,1\}$. Statement (i) implies $\tilde{\xi }_0\subset \tilde{\mathcal {O}}_n$ and $\tilde{\mathcal {O}}_n\setminus \tilde{\xi }_0$ is a topological disc. We claim that $\tilde{\xi }_1$ intersects $\tilde{\mathcal {O}}_n$. Let $\textsf {R}$ be the generator of the $\mathbb {Z}_2$-action on $\tilde{M^{\rho }}$. Note that $\tilde{\xi }_0\cup \tilde{\xi }_1$ is the singular locus of this group action. Given $p\in \tilde{\mathcal {O}}_n\setminus (\tilde{\xi }_0\cup \tilde{\xi }_1)$ we have $p\ne \textsf {R}p\in \tilde{\mathcal {O}}_n$. Since $\tilde{\mathcal {O}}_n\setminus \tilde{\xi }_0$ is connected, a curve $\gamma \subset \tilde{\mathcal {O}}_n\setminus \tilde{\xi }_0$ connects *p* and $\textsf {R}p$. If γ is disjoint from $\tilde{\xi }_1$ then $\gamma \cup \textsf {R}\gamma$ contains a simple closed curve winding around $\tilde{\xi }_1$. This curve is contractible in $\tilde{\mathcal {O}}_n\setminus \tilde{\xi }_0$ because $\tilde{\mathcal {O}}_n\setminus \tilde{\xi }_0$ is a topological disc. Therefore, $\tilde{\mathcal {O}}_n\cap \tilde{\xi }_1$ must be nonempty. Consequently, the intersection $\mathcal {O}_n\cap \xi _k$ is nonempty for all odd *k* as claimed.
(ii) We now investigate the orientability of $\mathcal {O}_n$ depending on *n*. Since the quotient $\tilde{\mathcal {O}}_n$ is a topological Möbius band, there exists a curve in $\partial \mathcal {O}_n$ connecting the start point of the segment $\xi _0$ (measured by distance from the origin) with the end point of the segment $\xi _2$ without intersecting any $\xi _k$ for $k\notin \{0,2\}$. This means that $\mathcal {O}_n$ makes an odd number *j* of half-twists between $\xi _0$ and $\xi _2$. Thus, $\mathcal {O}_n$ comprises *nj* half-twists in total, implying (ii). Alternatively one can argue as follows: The singular locus of the subgroup generated by $\textsf {R}_{e_1}^{\pi }\in \mathbb {D}_n$ acting on $M^{\rho }$ is given by $\xi _0\cup \xi _n$. Let *n* be odd and suppose that $\mathcal {O}_n$ is orientable. Then there exists a globally smooth choice of a unit normal vector field ν on $\mathcal {O}_n$. The map $\textsf {R}_{e_1}^{\pi }$ acts as an isometry on $\mathcal {O}_n$ and preserves ν at $\xi _n\cap \mathcal {O}_n$ because the intersection is orthogonal and nonempty as shown in (i). However, $\textsf {R}_{e_1}^{\pi }$ reverses ν on $\xi _0\subset \mathcal {O}_n$ which contradicts the assumption of orientability. Let now *n* be even. Then $\mathcal {O}_n$ contains both $\xi _0$ and $\xi _n$ by (i). Since $\mathcal {O}_n$ is either an annulus or a Möbius band, the set $\mathcal {O}_n\setminus (\xi _0\cup \xi _n)$ has two connected components $V_1$ and $V_2=\textsf {R}_{e_1}^{\pi }V_1$, both of them topological discs. Given a smooth unit normal vector field ν on $V_1$ we obtain a compatible unit normal vector field on $V_2$ by mapping -ν to $V_2$ via $\textsf {R}_{e_1}^{\pi }$. Therefore, $\mathcal {O}_n$ is orientable.
(iii) For the area estimate, we set $\ell =2(\rho -1)$ as in (15) and consider the surface $L_n:=\Phi _\ell ^{-1}(\mathcal {O}_n)$ which, by Lemma 2.2, is $\ell \pi /n$-periodic in $Z=\mathbb {B}^2\times \mathbb {R}$ containing $\zeta _k$ defined in (6) for every even *k*. It passes to a properly embedded surface $\tilde{L}_n$ in the quotient $\tilde{Z}_{\ell /n}$ as defined in (5). Moreover, $\tilde{L}_n$ has the same topology as the Möbius band $\tilde{\mathcal {O}}_n$. As in the proof of Lemma 3.4 we may apply Lemmata 3.3 and 2.1 to obtain
  24 $$\begin{aligned} \mathscr {H}^2(\mathcal {O}_n)&=n\mathscr {H}^2(\tilde{\mathcal {O}}_n)\ge n\mathscr {H}^2(\tilde{L}_n)\ge n\pi . \end{aligned}$$
  Since $m_1\mathscr {H}^2(\mathcal {O}_n)=W_\Pi \in ]n\pi ,2n\pi [$ by (22), estimate (24) implies $m_1=1$ and thus we have $n\pi<\mathscr {H}^2(\mathcal {O}_n)<2n\pi$ as claimed.

□

**Fig. 6:**
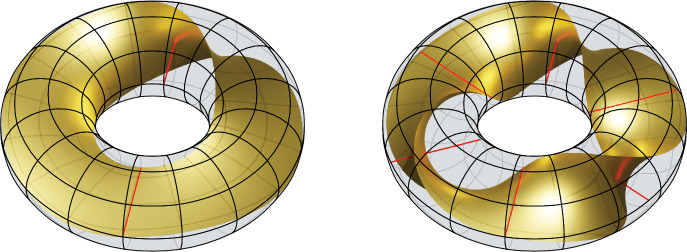
Free boundary minimal Möbius bands $\mathcal {O}_n\subset M^{\rho }$ with $n\in \{1,3\}$ half-twists

**Fig. 7:**
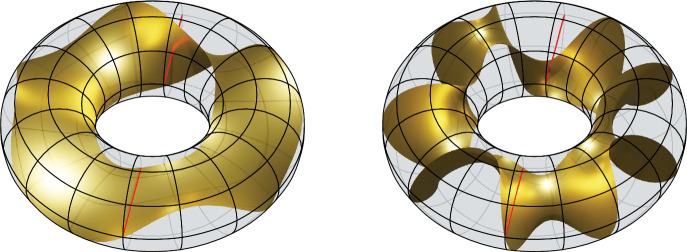
Nonuniqueness of $\mathbb {D}_1$-equivariant free boundary minimal Möbius bands in $M^{\rho }$

## Conjectures and simulations

We conjecture that the surfaces $\mathcal {O}_n\subset M^{\rho }$ constructed in Theorem 1.1 (i)–(ii) exist in fact for *any* number $n\in \mathbb {N}$ of half-twists. We visualise the conjectural surfaces for $n\in \{1,3\}$ in Fig. 6. However, we expect that the upper area bound in claim (iii) does require a lower bound on *n* as stated in the theorem. Moreover, if *n* is too small depending on the radius ρ, we expect the minimal surfaces $\mathcal {O}_n$ to have $\mathbb {D}_n$-equivariant index greater than 1. Their variational construction would thus require multi-parameter sweepouts, which are beyond the scope of this article.

**Fig. 8:**
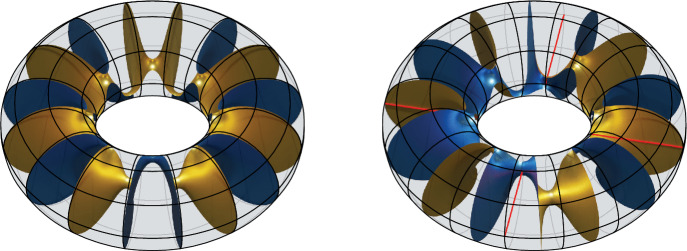
Free boundary minimal disc stackings in $M^{\rho }$ with 0 respectively 2 half-twists

Furthermore, it seems that – unlike discs – free boundary minimal annuli and Möbius bands in $M^{\rho }$ are surprisingly nonunique even if one prescribes their full symmetry group (see Fig. 7). Given any integer n≥0, there may exist *infinitely* many embedded solutions with *n* half-twists. Some inspiration for this conjecture comes from a method called “stacking” (cf. [4]). The simulations visualised in Fig. 8 suggest that a union of (sufficiently many) pairwise disjoint free boundary minimal discs in $M^{\rho }$ can be connected with *a single* half-catenoidal bridge between each pair of adjacent discs, and then perturbed into a free boundary minimal surface. (This might be surprising in view of [4], where the stacking construction requires *many* half-catenoidal bridges between each layer.) The relative positions of these bridges then determine the number *n* of half-twists.

## Data Availability

All data supporting this study are available within the article.

## References

[1] Almgren, F.: The theory of varifolds, mimeographed notes. , Princeton (1965)

[2] Bokowski, J., Sperner EJr.: Zerlegung konvexer Körper durch minimale Trennflächen. J. Reine Angew. Math. **311**(312), 80–100 (1979)

[3] Carlotto, A., Franz, G., Schulz, M.: Free boundary minimal surfaces with connected boundary and arbitrary genus. Camb J Math **10**(4), 835–857 (2022)

[4] Carlotto, A., Schulz, M.B., Wiygul, D.: Disc stackings and their Morse index. Adv. Nonlinear Stud. **25**(3), 756–806 (2025)

[5] Carlotto, A., Schulz, M.B., Wiygul, D.: Index growth not imputable to topology. Proc Amer Math Soc **153**(4), 1787–1801 (2025). 10.1090/proc/17151

[6] Carlotto, A., Schulz, M.B., Wiygul, D.: Spectral estimates for free boundary minimal surfaces via Montiel-Ros partitioning methods. Anal. PDE **18**(7), 1715–1768 (2025). 10.2140/apde.2025.18.1715

[7] Colding, T.H., De Lellis, C.: The min-max construction of minimal surfaces. In: Surveys in differential geometry, Vol. VIII (Boston, MA, 2002), Surv. Differ. Geom., vol 8. Int. Press, Somerville, MA, p 75–107 (2003)

[8] Colding, T.H., Minicozzi, W.P.I.I.: A course in minimal surfaces, Graduate Studies in Mathematics, vol. 121. American Mathematical Society, Providence, RI (2011)

[9] Courant, R.: The existence of minimal surfaces of given topological structure under prescribed boundary conditions. Acta Math. **72**, 51–98 (1940)

[10] De Lellis, C., Pellandini, F.: Genus bounds for minimal surfaces arising from min-max constructions. J. Reine Angew. Math. **644**, 47–99 (2010)

[11] Dierkes, U., Hildebrandt, S., Küster, A.: Minimal surfaces I, Grundlehren der mathematischen Wissenschaften, vol. 295, Springer-Verlag (1992). (boundary value problems)

[12] Franz, G., Schulz, M.B.: Topological control for min-max free boundary minimal surfaces. J. Eur. Math. Soc. (2023). arXiv:2307.00941 to appear

[13] Fraser, A., Sargent, P.: Existence and classification of -invariant free boundary minimal annuli and Möbius bands in . J. Geom. Anal. **31**(3), 2703–2725 (2021). 10.1007/s12220-020-00371-9

[14] Fraser, A., Schoen, R.: Uniqueness theorems for free boundary minimal disks in space forms. Int Math Res Not IMRN **17**, 8268–8274 (2015). 10.1093/imrn/rnu192

[15] Fraser, A., Schoen, R.: Sharp eigenvalue bounds and minimal surfaces in the ball. Invent. Math. **203**(3), 823–890 (2016)

[16] Freitag, E.: Complex Analysis 2. Universitext, Springer, Heidelberg (2011)

[17] Grüter, M., Jost, J.: On embedded minimal disks in convex bodies. Ann. Inst. H. Poincaré Anal. Non Linéaire **3**(5), 345–390 (1986)

[18] Haslhofer, R., Ketover, D.: Free boundary minimal disks in convex balls. J. Reine Angew. Math. **828**, 307–326 (2025). 10.1515/crelle-2025-0068

[19] Hatcher, A.: Algebraic topology. Cambridge University Press, Cambridge (2002)

[20] Jost, J.: Existence results for embedded minimal surfaces of controlled topological type. I. Ann Scuola Norm Sup Pisa Cl Sci (4) **13**(1), 15–50 (1986)

[21] Karpukhin, M., Kusner, R., McGrath, P., et al.: Embedded minimal surfaces in and via equivariant eigenvalue optimization. preprint arXiv:2402.13121

[22] Ketover, D.: Equivariant min-max theory. preprint (2016a) arXiv:1612.08692

[23] Ketover, D.: Free boundary minimal surfaces of unbounded genus. preprint (2016b) arXiv:1612.08691

[24] Ketover, D.: Genus bounds for min-max minimal surfaces. J Differential Geom **112**(3), 555–590 (2019)

[25] Li MMc: A general existence theorem for embedded minimal surfaces with free boundary. Comm Pure Appl Math **68**(2), 286–331 (2015)

[26] Lima, V.: Bounds for the Morse index of free boundary minimal surfaces. Asian J Math **26**(2), 227–252 (2022)

[27] Maggi, F.: Sets of finite perimeter and geometric variational problems, Cambridge Studies in Advanced Mathematics, vol. 135. Cambridge University Press, Cambridge (2012)

[28] Marques, F.C., Neves, A.: Min-max theory and the Willmore conjecture. Ann of Math (2) **179**(2), 683–782 (2014)

[29] Marques, F.C., Neves, A.: Existence of infinitely many minimal hypersurfaces in positive Ricci curvature. Invent. Math. **209**(2), 577–616 (2017)

[30] Meeks, W.H.I.I.I.: The classification of complete minimal surfaces in with total curvature greater than . Duke Math. J. **48**(3), 523–535 (1981)

[31] Meeks, W.H.I.I.I., Weber, M.: Bending the helicoid. Math. Ann. **339**(4), 783–798 (2007). 10.1007/s00208-007-0120-4

[32] Meusnier, J.: Mémoire sur la courbure des surfaces, (1785)

[33] Mira, P.: Complete minimal Möbius strips in and the Björling problem. J. Geom. Phys. **56**(9), 1506–1515 (2006). 10.1016/j.geomphys.2005.08.001

[34] Nitsche, J.C.C.: Stationary partitioning of convex bodies. Arch Rational Mech Anal **89**(1), 1–19 (1985)

[35] de Oliveira, M.E.G.G.: Some new examples of nonorientable minimal surfaces. Proc Amer Math Soc **98**(4), 629–636 (1986). 10.2307/2045740

[36] Petrides, R.: Non planar free boundary minimal disks into ellipsoids, (2023). arXiv:2304.12111 preprint

[37] Pitts, J.T.: Existence and regularity of minimal surfaces on Riemannian manifolds, Mathematical Notes, vol. 27. University of Tokyo Press, Tokyo, Princeton University Press, Princeton, N.J. (1981)

[38] Ros, A.: The isoperimetric problem. In: Global theory of minimal surfaces, Clay Math. Proc., vol 2. Amer. Math. Soc., Providence, RI, p 175–209 (2005)

[39] Schulz, M.B.: Equivariant free boundary minimal discs and annuli in ellipsoids, (2024). arXiv:2406.13465 preprint

[40] Smith, F.: On the existence of embedded minimal -spheres in the -sphere, endowed with an arbitrary Riemannian metric, (1982). (PhD thesis)

[41] Toro Cardona, C.A.: Non-existence of free boundary minimal Möbius bands in the unit three-ball. Proc Amer Math Soc **153**(5), 2185–2198 (2025). 10.1090/proc/17163

